# In Memoriam—Samuel Swan, M. D.

**Published:** 1894-01

**Authors:** Thomas Skinner

**Affiliations:** 25 Somerset Street, London, W.


### IN MEMORIAM—SAMUEL SWAN, M. D.

EDITOR OF THE HOMŒOPATHIC PHYSICIAN:—I do not feel inclined to allow the departure of my dear old friend to a better, a happier, more useful, and truer state of existence than any of us can possibly enjoy on this side of Time, without some small tribute to his memory, to his worth, as a pioneer of one of the greatest discoveries in therapeutics. Swan needs no floral wreaths on his bier, or strewed over his grave; he made, he wove his own wreaths by his provings of Luesinum, Medorrhinum, Lac-caninum, Lac-vacc., Lac-vacc-deflor., Lac-vacc-coag., Lac-felinum, etc., etc., etc.

Samuel Swan bravely encountered the crushing hatred of a body in the profession who considers itself *infallible*—the only authority as to what is reconcilable with the teachings of the master, and what is not—and what is not, is ISOPATHY. If such followers of the master live long enough, they may live to know that Samuel Swan was in the right, and that they were—well, I will not say where they were or are!

Samuel Swan was no more infallible that the rest or any of us—Thomas Skinner being no exception to the rule—not even excepting those who have joined the majority, such as Hering, H. N. Guernsey, Lippe, Dunham, and P. P. Wells—but Swan was unlike all of these men, he had an originality of thought, a gem which is priceless in any man; and it was the original course which he struck out for himself which gave us the possession of Tuberculinum, Septicæmia or Pyrogen, *et hoc genus omne*, to say nothing of the deep debt which the homœopathic profession owes him, for the introduction (along with Dr. Fincke) of the high attenuations in general by mechanical means.

As time advances, I make no doubt that Samuel Swan's name, minus his few faults, will be handed down to posterity second only to Hahnemann and Bœnninghausen, as an original and true thoughtsman.

Yours fraternally,

THOMAS SKINNER, M. D.,
25 Somerset Street, London, W.

November 19th, 1893.

